# Clinical Application of Standardized Cognitive Assessment Using fMRI. I. Matrix Reasoning

**DOI:** 10.3233/BEN-2008-0223

**Published:** 2009-07-29

**Authors:** Mark D. Allen, Alina K. Fong

**Affiliations:** ^1^Psychology DepartmentBrigham Young UniversityProvoUTUSA; ^2^Neuroscience CenterBrigham Young UniversityProvoUTUSA

**Keywords:** fMRI, matrix reasoning, cognitive assessment, normative data

## Abstract

Functional MRI is increasingly recognized for its potential as a powerful new tool in clinical neuropsychology. This is likely due to the fact that, with some degree of innovation, it is possible to convert practically any familiar cognitive test into one that can be performed in the MRI scanning environment. However, like any assessment approach, meaningful interpretation of fMRI data for the purpose of patient evaluation crucially requires normative data derived from a sample of unimpaired persons, against which individual patients may be compared. Currently, no such normative data are available for any fMRI-based cognitive testing protocol. In this paper, we report the first of a series of fMRI-compatible cognitive assessment protocols, a matrix reasoning test (f-MRT), for which normative samples of functional activation have been collected from unimpaired control subjects and structured in a manner that makes individual patient evaluation possible in terms of familiar z-score distributions. Practical application of the f-MRT is demonstrated via a contrastive case-study of two individuals with cognitive impairment in which fMRI data identifies subtleties in patient deficits otherwise missed by conventional measures of performance.

## 1. Introduction

Over the last decade and a half, functional magnetic resonance imaging (fMRI) has become, by far, the dominant research tool for studying the neural substrates of cognitive processes, both in normal subjects as well as in those with cognitive/brain impairments. It is not surprising then, that great interest should arise in potential clinical applications of fMRI as a diagnostic and assessment tool for neurologists, neuropsychiatrists, neuropsychologists, and any other clinicians who routinely evaluate brain functioning in individual patients. Indeed, recent policy developments by professional governing organizations such as Division 40 of the American Psychological Association [24] and the American Medical Association Current Procedural Terminology Editorial Panel [5,11] clearly indicate that these organizations anticipate increased demand for fMRI in clinical settings in the near future.

There are, however, several factors that currently limit the full utility fMRI as a diagnostic and assessment tool. These limitations, which primarily involve issues of validity and reliability, are articulated especially well in a recent paper by Brown [6]. One of the more significant problems that Brown identifies, with respect to the validity of clinical fMRI, is the absence of large-scale normative data sets derived from representative samples of individuals without brain impairment, which would allow quantitative evaluations of patient brain activation patterns for any given cognitive process, with respect to the population from which the patient comes. Without well structured normative data-bases that accurately characterize normal brain activation patterns, it is currently not possible to provide contextualized, quantitative assessments of individual patient outcomes, expressed in terms of statistical deviation from what would be expected in the absence of pathology.

ISSN 0953-4180/08/$17.00 © 2008 – IOS Press and the authors. All rights reserved

In this study, we describe a test protocol based on the classic Matrix Reasoning Test (MRT), designed specifically for use in the fMRI scanning environment. This MRT adaptation, along with an adaptation of a standard verbal fluency test reported in a companion paper (Allen and Fong, 2008), represent two of a series of six fMRI protocols we have developed with the potential for clinical application. Other protocols in this series include adaptations of such familiar tests as the Trail Making Test-B [29], Face Memory Test [30], Verbal Memory Test [8], and Picture Naming Test [14]. In order to maximize the interpretability and practical application of the normative data obtained from our fMRI assessment protocols, with respect to comparable normative data sets derived from conventional cognitive tests, we modeled our scanning protocols as closely as possible after the most commonly used neuropsychological tests in contemporary assessment batteries [17, 21,35]. The motivation to keep our fMRI protocols closely structured to commonly used versions of these familiar neuropsychological tests follows a statement given in Hart et al. [11], regarding the governance of procedures and billing codes for clinical fMRI:

> At present, no standardized cognitive/neuropsychologic testing packages have been certified by any duly charged governing body in terms of appropriate stimuli or tasks. Testing guidelines in terms of choice of stimuli, tasks, and performance parameters should thus presently conform in general to standard guidelines accepted for neurobehavioral and/or neuropsychologic testing.

Nevertheless, aside from some notable exceptions [22,23,27,32,38], few neuroimaging studies have been designed specifically to mimic neuropsychological exams.

There are three objectives of this paper. First we describe the development our fMRI adaptation of the MRT, which we have labeled the *f-MRT* protocol. This includes a description of the methods by which we generated our test stimuli, as well as an empirical comparison between performance on our stimuli and those from a comparable conventional version of the test – namely, the Raven's Colored Progressive Matrices (CPM). These concurrent validity measures include data from independent samples of both neurologically impaired and neurologically unimpaired subjects. Second, we present functional activation data from 32 control subjects without brain impairment who were scanned using the protocol. Finally, we introduce a method for assessing the reliability of activation patterns across subjects, which in turn provides the basis for a map of normative activation patterns for each exam. Specifically, each normative brain map represents a distribution of activation values within functionally-defined brain regions of interest (ROI), which allows one to evaluate individual patient result profiles, as expressed in *z-score*s, for any ROI or set of ROIs under investigation. An example of this ROI-based normative activation map, for the MRT data, will be presented here.

Following the experimental portion of this study, we will present a practical application of the f-MRT, where we describe and contrast fMRI testing outcomes of two patients suffering traumatic brain injury. The primary objective of that section will be to demonstrate the ability of the f-MRT to distinguish between subtypes of neurological impairments associated with otherwise indistinguishable performance on behavioral measures alone.

Concerning our first objective, there are many inherent challenges in converting a conventional "paper and pencil" test, such as the MRT, into a protocol that can be used to acquire valid and interpretable fMRI data. These challenges arise primarily from the physical and temporal limitations imposed by the MRI scanning environment. For example, during scan acquisition, subjects' heads must remain entirely immobilized, such that it is generally not possible to obtain overt vocal responses during scanning, except under certain limited circumstances (see for example [1,3,4,9,10,13]). Likewise, all other body movements must be kept to an absolute minimum, such that subject responses are largely limited to hand and finger actions (e.g., button presses). Another major challenge concerns the temporal constraints of the fMRI method. The use of a typical MRI scanning facility is time-based and expensive. This, along with other factors such as subject fatigue and boredom necessitates, in most cases, much shorter test durations for fMRI protocols than for the respective conventional tests they are modeled after. Other temporal constraints are more complex. For example, the fMRI technique critically relies on the detection of blood oxygen level dependent (BOLD) changes in the brain, which are governed by intricate physiological processes with narrowly constrained temporal parameters [20]. Therefore, it is usually necessary to place stricter control over exactly when and for how long a subject engages in a given cognitive activity or response behavior, compared to the restrictions found in comparable conventional tests.

Because of these and other constraints, developing an fMRI-compatible protocol, such as the MRT adaptation described here, requires a unique set of methods for presenting stimuli and obtaining subject responses in a manner which, to the extent possible, retains enough crucial similarities with each corresponding conventional test, to allow meaningful comparisons to be made between fMRI activation patterns and performance patterns on conventional tests. In this way, our protocols might qualify as appropriate "testing packages" as defined by Hart et al. [11]. In order to verify the assumption that we have accurately modeled a tradition MRT, however, we performed convergent validity tests (reported below) in which subjects were administered both the standard MRT test (the CPM) and the f-MRT, with both tests administered outside of the scanning environment.

Raven's Progressive Matrix (RPM) is widely regarded as a classic test of non-verbal reasoning in the visual modality [2,28]. The task employed in the RPM, referred generically as *matrix reasoning*, has been adapted for use in many prevalent assessments, including variants of the original RPM (i.e., the Colored and Advanced Progressive Matrices tests), as well as the Weschler Adult Intelligence Scale (WAIS) and its variants. It is commonly used to assess visually-based abstract problem-solving ability, while putatively placing little or no demands on verbal, motor, and complex visual processing skills [33]. The matrix reasoning task requires subjects to analyze visual pattern changes that occur along multiple dimensions within an array of stimulus objects. Subjects must ascertain the nature of each pattern change along each dimension independently, and then integrate this knowledge to determine the correct solution. Thus, the matrix test relies heavily on visuospatial working memory, goal/sub-goal processing, inductive reasoning, and deductive reasoning.

The first fMRI study to directly test neural activity associated with solving matrix reasoning problems [22] found activation predominantly in bilateral inferior and middle frontal gyri, as well as bilaterally throughout the dorsal visual processing stream (inferior/middle occipital cortex and inferior/superior parietal cortex). More recent studies [7,15] using somewhat improved technology and methods (e.g., more coverage of the dorsal brain) replicated this basic finding, with additional activations found in medial frontal cortex, including dorsal anterior cingulate and supplementary motor cortex, as well as greater extents of dorsolateral prefrontal cortex. Finally, a very recent study by Melrose et al. [19] identified reliable activation of the basal ganglia during performance of this task. Although the studies reported above vary somewhat in design, they each employed stimuli and protocols with the specific attempt to model familiar neuropsychological exams – namely the WAIS-III matrix reasoning subtest, and the original RPM. Given the similarities between our objectives and design approach, the results from these studies allow reasonable predictions about the activation patterns we expect to observe in this experiment, thus providing some means for assessing the validity of our protocol at the neural systems level.

## 2. Methods

### 2.1. Participants

Thirty-two participants (16 male, 16 female) between 20 and 39 years old (Mean = 25.04; S.D = 4.23) volunteered to serve as control subjects for this study. Participants received no compensation, but were told that they would have the opportunity to see structural and functional images of their brains after the study. Hand dominance was assessed using the Edinburgh Handedness Inventory [25]. All but two subjects (one male, one female) were determined to be dominantly right-handed. Mean L.Q. scores on the Edinburgh handedness scale – where scores above +48 suggest strong right handedness – were +71.8 (Decile R.3), sd = 35.0 for females; and +69.1 (Decile R.3), sd = 31.5 for males; with no significant difference between sexes ($t = 1.36$, $p > 0.1$). All subjects were Caucasian, except for one Hispanic woman and one Asian/Pacific Islander male, and spoke English as their first language.

All participants were determined to have no history of neurological impairments (assessed by a screening questionnaire), nor history of significant psychological pathology, and reported no use of psychotropic medications. High resolution 3D SPGR and $T_2$ axial FLAIR MRI scans revealed no detectible brain abnormalities in any control subjects, as determined by a qualified neuroradiologist. In addition to the overall good neuropsychological health of our control participants, they were also determined to be high functioning in cognitive ability. All subjects had completed at least one year of college education and were in good academic standing at a university with high admission/continuance standards. All participants consented to release pre-admission records of ACT (or SAT) scores. Analysis of mean scores (with SAT converted to ACT equivalents) revealed overall high performance, with a mean of 30 (sd = 4.30) for females, and 29 (sd = 2.16) for males, with no significant difference between sexes ($t = 1.38$, $p > 0.1$).

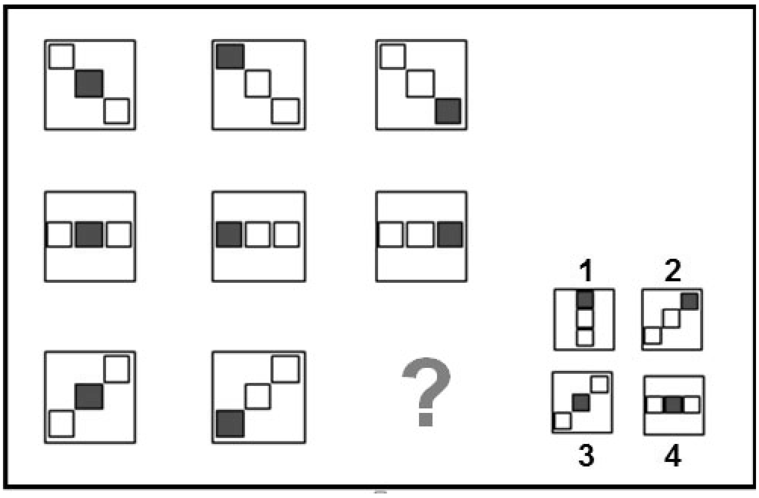

Fig. 1. Sample item from the f-MRT matrix reasoning test.

### 2.2. Materials

For this study, we created a set of 24 test stimulus items, conceptually modeled after problems found on the Raven's Progressive Matrices test, as well as items from the matrix reasoning subtest of the WAIS-III. It should be noted, however, that all items were uniquely devised by the authors of this study – no direct reproductions or replications of items from any existing copyrighted exams were used. These items, along with 24 alternate stimuli, and 4 practice items, are referred collectively as the f-MRT. Each stimulus consists of a $3 \times 3$ matrix of complex visual figures, with one figure missing (see Fig. 1).

For each matrix problem, participants were given the simple instruction to "indicate what the missing figure should be," and to then select it from among the four choice alternatives presented on the right side of the matrix. For example, given the problem in Fig. 1, the participant must discover that the linear orientation of internal boxes varies as a function of rows, whereas the position of the filled-in box varies as a function of columns. Thus, the correct choice is "2," where the orientation of boxes is directed upward from left to right, consistent with the other figures in the bottom row, and the filled-in box occupies the rightmost position, consistent with the other figures in third column. Following most standard applications of matrices tests, our participants were told to place more importance on response accuracy than on response speed. All participants were given 4 practice trials. After each practice trial, the participant was asked to explain her/his reasoning for solving the problem. If the participant made an incorrect choice, the experimenter revealed the correct choice and the reasoning behind selecting that choice.

One important consideration in the creation of our f-MRT stimuli concerns problem difficulty. In order to obtain a reliable fMRI signal from a single subject associated with a given cognitive task, it is necessary to take repeated measurements of signal change across several epochs in which the subject repeatedly performs that cognitive task. An optimal measurement, therefore, requires that the subject engage cognitive mechanisms in a manner that is as consistent as possible across repetitions of the task. This demand for consistency across task repetitions, however, leads to some difficulty in modeling familiar MRT protocols in the fMRI environment. Most conventional versions of the MRT (e.g., the RPM), include a range of difficulty across test items, where problems become increasingly more difficult as the test progresses. A straightforward adaptation of such a test, therefore, would lead to unacceptable levels of fMRI signal variability across task repetitions for reliable statistical modeling within a single subject.

The challenge, then, was to create an MRT protocol which would retain the most fundamental psychometric properties of the protocols practitioners are familiar with, while at the same time employing optimal fMRI design techniques. The strategy we chose was to use items of moderate difficulty, that is, matrix problems modeled after those found in the medium range of "progressive" style MRTs, such as the RPM and the WAIS-III. To be more specific, we estimate our stimuli to be about as difficult as items near the ends of sections A and B of the standard RPM. Alternatively, one might compare the f-MRT difficulty level to that of the Raven's Colored Progressive Matrices test (CPM), given that the difficulty of the CPM is equivalent to sections A and B of the standard RPM.

Because the f-MRT, by design, does not employ a "progressive" pattern of item difficulty, we cannot claim that it fully models the CPM, or any other single matrix test, in its entirety. Nevertheless, we might reasonably expect considerable correlations in performance between the f-MRT and the CPM. The reason for this is simply that subjects are more likely to make errors on the more difficult items of the CPM – that is, on those most similar to items on the f-MRT.

### 2.3. *Evaluation of concurrent validity: The f-MRT and Raven's Colored Progressive Matrices*

In order to assess correlations in performance between the f-MRT and the CPM, we collected additional data from a sample of 69 individuals without neurological impairment, as well as 17 individuals diagnosed with neurological/cognitive impairments. The 69 subjects without impairment were matched demographically to the participants in the fMRI study, in terms of age, sex, and education level. The 17 patients with neurological impairments included 6 women and 11 men with an age range of 22-85 years, who were referred for clinical fMRI scans. This sample included 1 individual diagnosed with vascular dementia, 4 with probable Alzheimer's Disease, and 12 with cognitive impairments following traumatic brain injury. All participants completed both the 36-item CPM and the 24-item f-MRT, where the f-MRT was converted into a "paper-and-pencil" format such that it could be administered exactly as the CPM is. Both tests were given in a single session with test-order counterbalanced across participants.

#### 2.3.1. *Unimpaired subjects*

Performance scores for this group of 69 subjects were very high on both tests. The mean percent correct for the CPM was 97.37 (sd = 3.48; range = 86–100), which is in nearly perfect agreement with the normative data reported by Yuedall et al. [37] for normal subjects in this age range. The mean percent correct for the f-MRT was 98.36 (sd = 2.98; range = 88–100). Correlation analysis revealed a coefficient of +0.90, suggesting that the f-MRT and CPM show strong concurrent/convergent validity in unimpaired populations.

#### 2.3.2. *Neurologically impaired subjects*

Performance scores for this group of 17 subjects showed substantially more variability. The mean percent correct for the CPM was 86.11 (sd = 15.74; range = 47–100). The mean percent correct for the f-MRT was 87.25 (sd = 14.62; range = 50–100). Correlation analysis revealed a coefficient of +0.88, suggesting that the f-MRT and CPM show strong concurrent/convergent validity in a sample of patients with a variety of neurological/cognitive impairments.

### 2.4. *fMRI testing procedures*

All test stimuli were presented via MRI-compatible LCD goggles. Participants were instructed to make their responses by pressing one of four buttons on a fiber-optic response pad, held in both hands, using the index and middle fingers of each hand. At the beginning of each session, a "please wait prompt" appeared for 8 seconds to allow for T1 relaxation effects, followed by a 2-second "Ready?" prompt, after which the first test stimulus appeared. With each button-press response, the participant's accuracy and latency were recorded and the computer display was advanced to the next task epoch. Subjects were given the opportunity to solve as many of the 24 problems as possible during a functional scanning session of 4 minutes. Pilot trials revealed a consistent response time range of 1–4 seconds for most items. Each test epoch, consisting of one test item, alternated with an 11-second "rest" epoch, in which subjects were instructed to count covertly from 1 to 10. This simple counting task is recommended as an optimal minimal-demand cognitive activity for "rest" epochs in fMRI experiments [34]. Thus, the duration of each test epoch varied, depending on subject response time to a given test item, whereas rest epochs were fixed at 11 seconds.

## 3. Data analysis

### 3.1. *Image acquisition*

Functional images were acquired with a 1.5-T GE scanner at 23 contiguous axial locations with a slice thickness of 5 mm, using an EPIBOLD sequence with the critical parameters TR = 2000; TE = 40 ms. Conventional pre-processing and statistical analyses were performed using MRIcro and SPM2 (http://www.fil.ion.ucl.ac.uk) software packages, respectively. Preprocessing procedures included acquisition time realignment, using sinc interpolation, followed by motion correction with EPI distortion unwarping. No head movement exceeded 1 mm translation or $1^\circ$ rotation displacement. After motion/distortion correction, all functional volumes were spatially normalized and resampled using the Montreal Neurological Institute (MNI) templates implemented in SPM2, and spatially smoothed with an 8 mm FWHM Gaussian kernel, in order to increase signal-to-noise ratio and to reduce the effects of moderate inter-subject variability in brain anatomy. A high-resolution 3D SPGR whole-head volume was also collected from each subject and examined by a neuroradiologist for any structural anomalies that might disqualify the participant as a "normal" control subject. Each subject's SPGR image was then coregistered and normalized to their mean functional image in order to perform subject-specific ROI analyses that take into account individual variability in cortical landmark organization.

### 3.2. Conventional fMRI analyses

#### 3.2.1. Subject-level analysis

A time-series ANCOVA implemented in SPM2 was used to test each voxel, for each subject, against the null-hypothesis that changes in BOLD signal in that voxel, over the duration of the experiment, did not significantly correlate with the temporal sequencing of the cognitive task of interest. A boxcar waveform convolved with a synthetic hemodynamic response function (HRF) with a 4 sec lag-to-peak was used to model task-related activation. The data were high-passed-filtered in time, using a set of discrete cosine basis functions with a cut-off period of 128 seconds, and conditioned for temporal autocorrelations using AR1 correction. For each participant, $t$-values for the contrast test condition versus control condition, as well as the simple contrast test condition (against an implicit baseline) were computed for each voxel, using the parameter estimates of the ANCOVA. The resulting 3-dimensional contrast map from each subject was saved for further subject-level ROI analysis as well as for random effects (RFX) group-level analysis.

#### 3.2.2. Group-level analysis

Activation at the group level was analyzed using the RFX approach recommended by Penny et al. [26], in which the value of the sum of the contrast weights for each voxel from each subject's ANCOVA was entered as a single data point in a *second-level* $t$-statistic computation, with the mean value for each voxel across subjects modeled as the effect term and the variance between subjects modeled as the error term. Significant activation peaks at the group-level are reported with a critical FWE corrected $p$-value of $<$0.001, and a voxel cluster extent threshold of 8.

### 3.3. ROI analysis

In addition to the RFX group-level analysis, we performed ROI-based analyses for each control participant. There were two reasons for this: First, as stated above, a primary objective of this study is to develop a method for assessing the reliability of activation patterns across subjects, in order to provide quantitative estimates of deviation for any individual patient against a normative sample. For our approach, these analyses were carried out in terms of functionally motivated ROIs. Second, the RFX model is sensitive only to activation that reliably occurs across subjects within in a relatively tight spatial proximity. Therefore, for some brain regions, such as dorsolateral prefrontal cortex, the RFX model might not be sensitive to some locally idiosyncratic, yet globally systematic patterns of activation. This may be due to the relatively larger size of these functional regions, as well as the complexity of the processes they support. For example, large functional regions in the dorsolateral prefrontal cortex in particular have been shown to display broad divergence of activation peaks across subjects, as well as variable foci within even single subjects over repetitions of the same task [18].

Our procedure for establishing functional ROI designations follows the automated anatomical labeling (AAL) parcellation scheme described by Tzourio-Mazoyer et al. [36]. Although the AAL program itself (e.g., as implemented in SPM2) is designed to operate within the space of MNI-normalized brains, its anatomical specifications for region boundary identification are explicit and comprehensive enough that they can be applied to individual (non-normalized) brains with reasonable precision. For our purposes, however, it was necessary to make a few supplemental designations within the standard AAL scheme. These modifications were motivated primarily from empirical observations, in terms of reliable activation patterns identified throughout our data sets, but were also justified on *a priori* grounds, in terms of functional sub-regions that have been reliably identified by neurophysiological studies.

Accordingly, we designated the following minor supplements and modifications to the AAL ROI cortical parcellation scheme:

1. A division between the superior and inferior portions of the precentral gyrus.
2. A merging of the posterior portions of the superior and middle frontal gyri into a single region corresponding to Brodmann's area 6, often labeled *premotor* cortex [31].
3. Designation of the *frontal operculum*, as the opercular portions of inferior frontal cortex and anterior insula.
4. Designation of the *frontal pole*, as the portions of middle and superior frontal gyri anterior to *pars triangularis*, or approximately anterior to the MNI y-axis plane coordinate y = +40.

After ROI parcellation of each subject's anatomical image, individual functional activation maps (with a single $t$-value assigned to each voxel) were overlain for analysis of activation peak distributions on a subject-by-subject basis. Prior to analysis, each $t$-map was smoothed with a 1.5 mm FWHM Gaussian spatial filter in order to condition extreme outlier $t$-value spikes within peak clusters. Each ROI was then inspected for the presence of cluster peaks. If an independent peak was found, the maximum (smoothed) $t$-score was extracted and saved as a data point for the group analysis of that ROI. If the maximum value within an ROI belonged to a cluster with a centroid in an adjacent ROI (i.e., the highest intensity voxel fell at the border of an adjacent ROI), the ROI was determined to not have a peak. When more than one peak was identified in an ROI, the locations of the peaks were catalogued and, if consistently found across subjects, used to motive further ROI divisions (e.g., list-item 1 above).

Following the above guidelines and procedures for extracting $t$-values from each ROI within each subject, means and standard deviations of extracted scores were computed across subjects and used to derive a normalized distribution of $z$-scores for each ROI.

## 4. Results

### *4.1. Task performance on the f-MRT*

The average response accuracy was 98% (range 92–100%). The mean reaction time for all participants across all trials was 3509 ms (SD = 1208 ms).

### *4.2. Group-level BOLD activation*

Significant activation at the group level (see Fig. 2) was highly consistent with patterns found in similar studies [7,15,19,27]. Results based on the contrast test-control differed only slightly from the simple contrast test (versus implicit baseline). Given our interest in whole brain activation associated with all perceptual and cognitive components of the MRT task we modeled, we report the slightly more comprehensive pattern found for the simple contrast test. See Table 1a for a complete summary. Consistent with the dominant visuospatial nature of this task, strong clusters of activation were found throughout the ventral and dorsal visual processing streams, extending from fusiform cortex, through inferior and middle occipital cortex, reaching the parietal lobe with foci in intraparietal sulcus. Strong bilateral precentral gyrus/premotor activation is also present and is most readily attributed to manual motor response preparation, but is also consistent with studies demonstrating activation in these areas for tasks involving abstract spatial processing [16,31]. Clear activation was present in frontal areas involved with problem solving, such as medial SMA/dorsal anterior cingulate, posterior areas of dorsolateral prefrontal cortex, and inferior frontal cortex. As predicted by sensitivity limitations of the RFX approach, though, dorsolateral prefrontal activation was not as robust in other areas strongly associated with problem solving, such as superior/middle frontal gyri and the frontal pole. However, the ROI analyses reported below clearly confirmed expected activation in these areas.

### *4.3. ROI analysis*

For all significant regions identified by the RFX model, independent activation peaks were confirmed in corresponding subject-specific ROIs, with very few exceptions at the $t$-value corresponding to the probability threshold $p < 0.001$, uncorrected (t > 3.45), and with no exceptions at the $t$-value corresponding to the threshold $p < 0.01$, uncorrected (t > 2.49). As for individual activation peaks found in ROIs that did not reach significance on the RFX model, the following was observed: There were a few spurious ROI peaks, which occurred infrequently (1 additional peak found in 2 subjects), which were not consistent across subjects. However, there were two regions in which significant peaks were found consistently across all subjects at the threshold $p < 0.001$, uncorrected (t > 3.45). These were bilateral superior/middle frontal gyrus, and the frontal pole (see Table 1b).

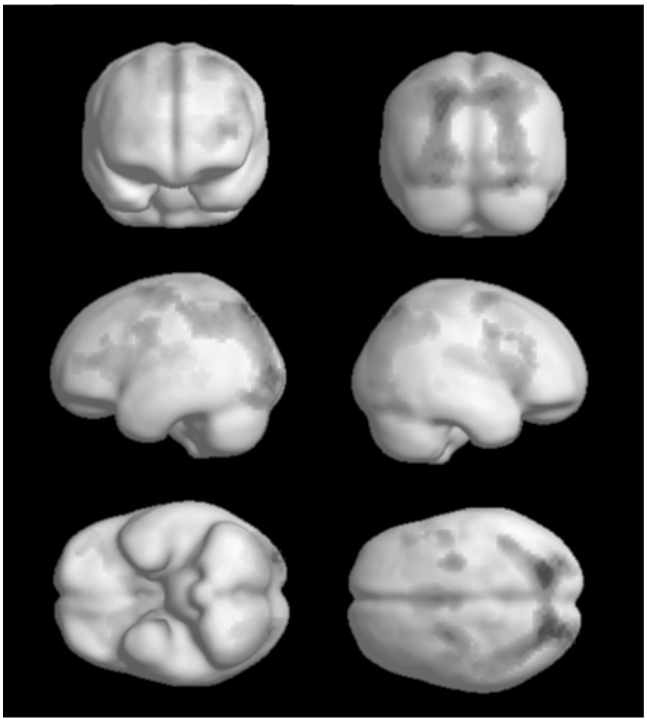

Fig. 2. Distribution of significant RFX-modeled group-level activation for the f-MRT matrix reasoning test. Surface-projection rendered on smoothed MNI brain template.

## 5. Summary and discussion of experimental results: Two methods of analysis

In this section, we described and tested methods for collecting fMRI data using a protocol that approximates traditional matrix reasoning tests as performed in conventional neuropsychological settings. The results from this experiment were highly consistent with results from previous studies that employed similar, though not identical, MRT protocols. Additionally, we performed individual ROI analyses on each control subject, using an augmented AAL parcellation scheme. This analysis revealed two important details: First, activation peaks were confirmed on a subject-by-subject basis for each significant region identified by the group-level RFX analysis, with nearly perfect consistency across subjects. Second, for the relatively large dorsolateral prefrontal and frontal pole regions, the ROI analyses revealed consistently large peaks in each subject. Because the precise location of these peaks varied across subjects by a few millimeters, however, this regional-level consistency was not revealed by the RFX analysis.

The second objective of our ROI analysis was to derive a distribution of sample $t$-values for each region activated in control subjects while performing the f-MRT. These regional distributions, in turn, allow one to perform quantitative assessments of individual subjects/patients who are administered this fMRI-adapted MRT protocol. Altogether, our analyses identify 11 critical ROIs for the f-MRT, including 9 regions identified by RFX and ROI analyses, as well as 2 additional frontal regions identified by ROI analysis alone. These regions are presented in Table 2, along with the means and standard deviations computed from the maximal (filter-conditioned) $t$-scores extracted from each of the 32 participants.

The data summary presented in Table 2 is most informative for two reasons. First, it allows one to assess the relative intensity of activation elicited in each ROI during average performance of the MRT task. This, in turn might suggest the relative contribution of each area in terms of successful task performance. Conclusions of this sort, of course, must be drawn with some caution, given that some cortical regions are inherently prone to higher BOLD signal detection, all things being equal. A second source of information concerns the variance of peak activations across individuals, and its application for clinical assessment. The variance within each ROI, as a numerical expression of the “normal”

Table 1a
Group-level random effects model activation foci ($p < 0.001$, FWE corrected) for the f-MRT matrix reasoning test

| Region (AAL) | MNI (x,y,z) | $t$-score |
|---|---|---|
| Fusiform/Inferior occipital cortex | | |
| Right | 35, −72, −16 | 15.74 |
| Left | −28, −84, −16 | 7.53 |
| Middle occipital gyrus | | |
| Right | 31, −89, 3 | 13.73 |
| Left | −28, −93, 2 | 10.62 |
| Superior parietal lobule/intraparietal sulcus | | |
| Right | 24, −72, 52 | 12.93 |
| Left | −36, −60, 48 | 12.00 |
| Medial supplementary motor area/ | | |
| Dorsal anterior cingulate gyrus | 1, 16, 45 | 11.79 |
| Thalamus | | |
| Right | 8, −21, 12 | 9.92 |
| Left | −10, −23, 13 | 11.26 |
| Precentral gyrus/Premotor area* | | |
| Superior | 27, −9, 61 | 9.09 |
| Right | −27, −9, 59 | 8.38 |
| Left | 46, −2, 39 | 8.93 |
| Inferior | −50, 2, 34 | 10.94 |
| Right | | |
| Left | | |
| Anterior insula/frontal operculum | | |
| Right | 37, 22, 8 | 9.11 |
| Left | −32, 26, 3 | 9.06 |
| Basal ganglia (caudate) | | |
| Right | 14, 12, 10 | 8.17 |
| Left | −10, 10, 11 | 8.89 |
| Inferior frontal gyrus | | |
| Right | 47, 28, 22 | 7.40 |
| Left | −47, 38, 13 | 8.93 |

*Premotor area = Portions of superior and middle frontal gyri corresponding to BA 6.

Table 1b
Subject-level analysis of the f-MRT. Additional activation foci present in all 32 control participants identified by ROI analysis

| Region (AAL) | Average $t$-score (SD) |
|---|---|
| Bilateral superior/middle frontal gyrus* | 8.42 (2.40) |
| Bilateral frontal pole** | 5.73 (2.00) |

*Excludes portions corresponding to BA 6.
**Portions of superior and middle frontal gyri anterior to y = +40 (MNI).

range of expected peak values, provides the statistical basis for evaluating activation patterns from individual subject/patients, with respect to group norms. A practical approach for applying this procedure is described in the next section.

## 6. Clinical application of the ROI analysis

The procedure for assessing a single patient's functional activation on the f-MRT (or any of the other fMRI-adapted neuropsychological protocols we have developed) with respect to our normative sample is fairly straightforward. Following the same methods applied to the control subjects, each patient's structural brain scan is first parcellated according to our augmented AAL scheme, and coregistered with his/her mean functional image. Next, maximal $t$-scores are extracted from each ROI of the patient's spatially filtered activation map, according to the same parameters set for the control subjects. Using the means and standard de-

Table 2
Means and standard deviations of extracted maximum t-values for each critical ROI associated with the Matrix Reasoning Test for a sample of 32 control subjects

| | Cortical regions | | | | | | | | | | |
|---|---|---|---|---|---|---|---|---|---|---|---|
| | Fusiform inferior occipital cortex | Middle occipital gyrus | Superior parietal lobule/ (IPS) | mSMA dorsal anterior cingulate | Thalamus | Precentral gyrus | Anterior insula | Superior/ middle frontal gyrus | Basal ganglia | Inferior frontal gyrus | Frontal pole |
| Mean | 10.02 | 9.78 | 10.67 | 8.82 | 4.84 | 9.54 | 5.82 | 8.42 | 5.35 | 7.29 | 5.73 |
| StDev | 3.24 | 3.92 | 3.55 | 3.09 | 1.71 | 3.17 | 1.81 | 2.40 | 1.58 | 1.94 | 2.00 |

IPS = Intraparietal Sulcus; mSMA = Medial Supplementary Motor Area.

viations from the control sample, a patient's maximal $t$-value can then be expressed as a $z$-score within each ROI.

This application highlights one of the ways in which fMRI assessments might greatly enhance knowledge about a patient's deficit with respect to a given cognitive task. For example, following the standard paper and pencil application of a matrix reasoning test, the clinician learns only whether a patient solves matrix problems correctly or not. With functional imaging, on the other hand, the clinician may learn further details about the functionality of the independent neural mechanisms that contribute to a patient's overall level of performance, as typically expressed in terms of a single value, score, label, or index (e.g., a $z$-score).

Since its development, we have analyzed over 100 patients with complaints of cognitive impairment using the f-MRT and its companion protocols. We present here sample data from two patients suffering moderate cognitive impairments following traumatic brain injury. Although these two patients share many commonalities, both in terms of overall cognitive functioning, as well as performance specifically on matrix reasoning tests, their f-MRT analyses show divergent profiles.

### 6.1. Patient descriptions

Descriptive summaries of the two patients of interest, referred to as P01 and P02, are given in Table 3. Both patients are female college students in their twenties who were involved in motor vehicle accidents. Both patients reported symptoms of persistent post-concussive syndrome, including difficulties with attention, verbal memory, verbal fluency, mood regulation, and motivation. Neuroradiological analyses were performed at the time of neuropsychological/fMRI assessment. Inspection of MRI brain images, including axial T1, axial FLAIR, and axial T2 FSE sequences, revealed no abnormalities. Comprehensive neuropsychological batteries administered at the time of fMRI scanning indicated global cognitive performance in the low-normal range for each patient's age, gender, and level of education (RBANS scores in Table 3 are representative in this regard). Of particular interest, both patients showed notable impairments on matrix reasoning tests – namely the CPM and the matrix reasoning subtest of the WAIS-III – with scores on both tests falling in the range of 1.5–2.5 standard deviations below normal.

### 6.2. Patient f-MRT analysis

Both patients were administered the f-MRT within two weeks of the time standard neuroimaging and neuropsychological assessments were administered. As indicated in Table 3, accuracy and reaction time performance by P01 and P02 on the f-MRT was consistent with performance on conventional tests, with scores in the range of $-0.61$ and $-1.93$ standard deviations below means for the control group.

#### 6.2.1. ROI analysis: Comparison of P01 and P02

The greatest value of the f-MRT assessment is that it provides information about patient functioning that goes beyond simple measures of test performance. ROI analyses of f-MRT data from P01 and P02 provide a case-in-point. Outcomes of the ROI analyses for P01 and P02 are given in Figs 3 and 4, respectively. It should be recalled that the values in these figures do not merely represent levels of functional activation for each ROI, but rather represent degree of statistical deviation from normal activation within each region.

Inspection of these figures reveals clear distinctions between P01 and P02. For example, the analysis for P01 in Fig. 3 implicates a disruption primarily of dorsolateral pre-frontal structures, where $t$-values fall around 2 standard deviations below the mean, as well as medial prefrontal cortex, where values fall more than 1 standard deviation below the mean. It appears then, that for P01, structures involved in the executive operations associated with the MRT task, including the dorsal anterior cingulate, appear selectively compromised.

Table 3

Demographic information and selection of neurobehavioral characteristics of sample patients P01 and P02. At the time of testing, both patients were concurrently administered conventional neuropsychological tests and the f-MRT

| | P01 | P02 |
|---|---|---|
| Sex | F | F |
| Age | 26 | 22 |
| Years of education | 16 | 14 |
| Time since injury (months) | 24 | 10 |
| Glasgow coma scale | 14 | Not reported |
| Loss of consciousness (minutes) | $<30$ | $<5$ |
| Post traumatic amnesia (hours) | | |
| Anterograde | 12 | 8 |
| Retrograde | 24 | None |
| RBANS total scale score (percentile) | 89 (23) | 94 (34) |
| Matrix reasoning subtest WAIS-III | | |
| Raw score (age percentile) | 10/24 (7) | 9/24 (6) |
| RCPM raw score (*$z$-score) | 32/36 ($-2.32$) | 33/36 ($-1.52$) |
| f-MRT performance (z-score) | | |
| Accuracy | 92% ($-1.92$) | 96% ($-0.61$) |
| Mean reaction time (ms) | 4807 ($-1.07$) | 5842 ($-1.93$) |

Notes: RBANS = Repeatable Battery for the Assessment of Neuropsychological Status; WAIS = Wechler Adult Intelligence Scale; RCPM = Ravens Coloured Progressive Matrices; *Based on Yeudall et al. [37].

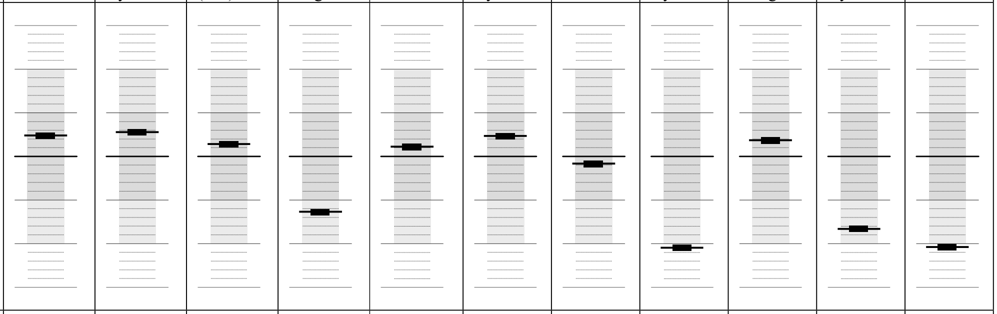

| Region | Fusiform/ Inferior Occipital Cortex | Middle Occipital Gyrus | Superior Parietal Lobule/ (IPS) | mSMA/ Dorsal Anterior Cingulate | Thalamus | Pre-central Gyrus | Anterior Insula | Superior/ Middle Frontal Gyrus | Basal Ganglia | Inferior Frontal Gyrus | Frontal Pole |
|---|---|---|---|---|---|---|---|---|---|---|---|
| Group Mean | 10.02 | 9.78 | 10.67 | 8.82 | 4.84 | 9.54 | 5.82 | 8.42 | 5.35 | 7.29 | 5.73 |
| Group StDev | 3.24 | 3.92 | 3.55 | 3.09 | 1.71 | 3.17 | 1.81 | 2.40 | 1.58 | 1.94 | 2.00 |
| Patient *t* value | 11.63 | 12.10 | 11.65 | 5.05 | 5.20 | 10.90 | 5.50 | 3.41 | 5.95 | 4.09 | 1.67 |
| Patient *z*-score | 0.50 | 0.59 | 0.28 | -1.23 | 0.21 | 0.43 | -0.18 | -2.10 | 0.38 | -1.65 | -2.03 |

IPS = Intraparietal Sulcus; mSMA = Medial Supplementary Motor Area

Fig. 3. Sample report of selected patient outcome (P01) on the ROI analysis of the f-MRT. Tick marks along vertical columns indicate patient $z$-scores ($-3$ to $+3$) for each brain region. Note: value levels do not simply represent relative activation in each region. Rather, they represent degree of statistical deviation from normal for each region.

In contrast to this, P02 shows a different pattern of activation deficits, where abnormalities are restricted primarily to parietal cortex and basal ganglia, where $z$-scores fall more than two standard deviations below the mean. This suggests that for P02, disruptions to mechanisms of spatial processing, and perhaps working memory processing capacity, provide the most likely explanation for her performance impairments on the MRT.

The contrastive outcomes for P01 and P02, then, illustrate that fMRI analysis offers a means for identifying differing patterns of disruption to the neurocog-

| Region | Fusiform/ Inferior Occipital Cortex | Middle Occipital Gyrus | Superior Parietal Lobule/ (IPS) | mSMA/ Dorsal Anterior Cingulate | Thalamus | Pre-central Gyrus | Anterior Insula | Superior/ Middle Frontal Gyrus | Basal Ganglia | Inferior Frontal Gyrus | Frontal Pole |
|---|---|---|---|---|---|---|---|---|---|---|---|
| |  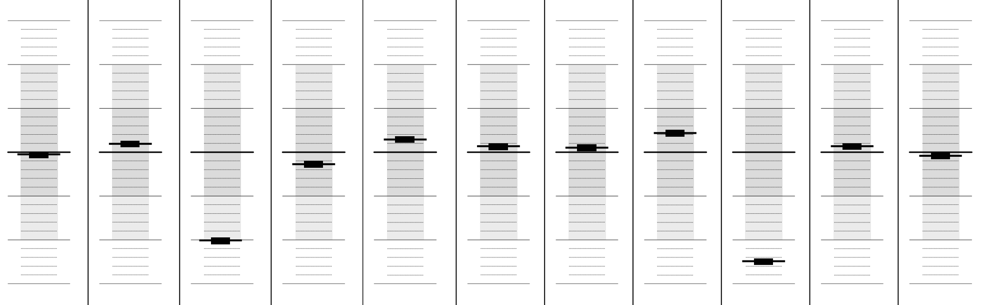 | | | | | | | | | | |
| Group Mean | 10.02 | 9.78 | 10.67 | 8.82 | 4.84 | 9.54 | 5.82 | 8.42 | 5.35 | 7.29 | 5.73 |
| Group StDev | 3.24 | 3.92 | 3.55 | 3.09 | 1.71 | 3.17 | 1.81 | 2.40 | 1.58 | 1.94 | 2.00 |
| Patient *t* value | 9.71 | 10.56 | 3.48 | 7.98 | 5.35 | 10.02 | 6.06 | 9.89 | 1.66 | 7.60 | 5.49 |
| Patient *z*-score | -0.09 | 0.20 | -2.02 | -0.27 | 0.30 | 0.15 | 0.13 | 0.61 | -2.33 | 0.16 | -0.12 |

IPS = Intraparietal Sulcus; mSMA = Medial Supplementary Motor Area

Fig. 4. Sample report of selected patient outcome (P02) on the ROI analysis of the f-MRT. Tick marks along vertical columns indicate patient $z$-scores ($-3$ to $+3$) for each brain region.

nitive systems that underlie a particular cognitive task across patients who otherwise show equal performance on behavioral measures alone.

## 7. Summary and conclusion

In this study we addressed the emerging need to provide neuropsychological assessments adapted for use with fMRI technology. We have presented here an adaptation of the Matrix Reasoning Test, the f-MRT, and shown that in a sample population of normal subjects, activation patterns are consistent both with previous fMRI studies using similar protocols, and activation is consistent with cognitive mechanisms hypothesized to be critical for successful performance on the MRT from a neural systems point of view. As such, we suggest that the data presented here represent a major step toward the full utility of fMRI as a diagnostic and assessment tool, specifically in that we provide a protocol that is structured in a way that allows performance and activation patterns to be compared in a meaningful way to performance on conventional MRT assessments familiar to most clinicians (e.g., as illustrated by the sample reports in Figs 3 and 4).

Another significant aspect of our current effort, though, is that we present a method for collecting and archiving activation patterns from normal subjects, such that the reliability of strength and locations of activation peaks can be evaluated on a region-by-region basis across subjects. This, in turn, allows one to determine a range of expected "normal" activation values for each region, and thus provides a normative scale for evaluating individual patient outcomes. Using this approach, we have demonstrated at least one case in which fMRI analysis revealed critical distinctions between two patients with highly similar performance abilities. It may not be surprising to find such distinctions for a complex task such as the MRT, where successful performance is hypothesized to rely on multiple cognitive sub-systems. While the goal of the current study has been to model a familiar version of the MRT, one might obtain even greater analytic specificity using fMRI, by examining the independent cognitive components that make up a complex cognitive task like the MRT in relative isolation, as described, for example, in the event-related fMRI approach to the Wisconsin card sorting task by Monchi et al. [22].
